# Treatment of avascular necrosis of the humeral head – Postoperative results and a proposed modification of the classification

**DOI:** 10.1186/s12891-022-05338-1

**Published:** 2022-04-27

**Authors:** Jonas Schmalzl, Annika Graf, Michael Kimmeyer, Fabian Gilbert, Christian Gerhardt, Lars-Johannes Lehmann

**Affiliations:** 1 Department of Trauma, Hand, Plastic and Reconstructive Surgery, Julius-Maximilians-University Wuerzburg, Oberduerrbacher Straße 6, 97080 Wuerzburg, Germany; 2 Department of Traumatology, Hand Surgery and Sports Medicine, St. Vincentius Clinic, Teaching Hospital Albert-Ludwigs-University Freiburg, Karlsruhe, Germany; 3 Medical Faculty Mannheim, Karls-Ruprecht-University Heidelberg, Mannheim, Germany; 4 Musculoskeletal University Center, Ludwigs-Maximilians-University Munich, Munich, Germany

**Keywords:** Proximal humeral fracture, Reverse shoulder arthroplasty, Anatomic shoulder arthroplasty, Fracture sequelae

## Abstract

**Background:**

Avascular necrosis of the humeral head after proximal humeral fracture i.e. type 1 fracture sequelae (FS) according to the Boileau classification is a rare, often painful condition and treatment still remains a challenge. This study evaluates the treatment of FS type 1 with anatomic and reverse shoulder arthroplasty and a new subclassification is proposed.

**Methods:**

This single-center, retrospective, comparative study, included all consecutive patients with a proximal humeral FS type 1 treated surgically in a four-year period. All patients were classified according to the proposed 3 different subtypes.

Constant score (CS), Quick DASH score, subjective shoulder value (SSV) as well as revision and complication rate were analyzed. In the preoperative radiographs the acromio-humeral interval (AHI) and greater tuberosity resorption were examined.

**Results:**

Of 27 with a FS type 1, 17 patients (63%) with a mean age of 64 ± 11 years were available for follow-up at 24 ± 10 months. 7 patients were treated with anatomic and 10 with reverse shoulder arthroplasty. CS improved significantly from 16 ± 7 points to 61 ± 19 points (*p* < 0.0001). At final follow-up the mean Quick DASH Score was 21 ± 21 and the mean SSV was 73 ± 21 points. The mean preoperative AHI was 9 ± 3 mm, however, 8 cases presented an AHI < 7 mm. 4 cases had complete greater tuberosity resorption.

The complication and revision rate was 19%; implant survival was 88%.

**Conclusion:**

By using the adequate surgical technique good clinical short-term results with a relatively low complication rate can be achieved in FS type 1. The Boileau classification should be extended for fracture sequelae type 1 and the general recommendation for treatment with hemiarthroplasty or total shoulder arthroplasty has to be relativized. Special attention should be paid to a decreased AHI and/or resorption of the greater tuberosity as indirect signs for dysfunction of the rotator cuff. To facilitate the choice of the adequate prosthetic treatment method the suggested subclassification system should be applied.

## Introduction

Avascular necrosis of the humeral head after proximal humeral fracture is a rare, often painful condition and treatment still remains a challenge. Boileau et al. [1, 2] classified the posttraumatic sequelae of proximal humeral fractures into four types. Fracture sequelae (FS) type 1 are defined as osteonecrosis of the humeral head or cephalic collapse. Up to date, several treatment options reaching from greater tuberosity osteotomy, hemiarthroplasty, anatomic total shoulder arthroplasty and reverse shoulder arthroplasty (RSA) have been published [3–6]. Anatomic shoulder replacement has shown promising midterm outcomes for FS type 1 [1, 2, 7]. Later on, worse results have been reported in patients with varus malunion and fatty infiltration of the rotator cuff and in this context a subclassification was suggested by Moineau et al. [5]. Under these circumstances anatomic shoulder arthroplasty (ASA) is not suitable and RSA has been shown to be a reliable treatment option [4, 6].

The purpose of this study was to report our results for the treatment of FS type 1 with anatomic and reverse shoulder arthroplasty and to retrospectively analyze prognostic factors that lead to unfavorable postoperative results in order to create a clinically reliable subclassification.

## Materials and methods

### Study design

This was a single-center, retrospective, comparative study. All consecutive patients with a proximal humeral fracture sequelae type 1 according to Boileau [1] treated in our institution between 2014 and 2018 were included. Exclusion criteria were neurological comorbidities and noncompliance with the postoperative rehabilitation protocol. The methods are similar to an article concerning fracture sequelae type 2 published recently by the corresponding author and therefore, there are overlapping passages in the methods section [8].

### Compliance with ethical standards

Institutional review board approval was obtained prior to commencing the study by the ethics committee of the Medical Faculty Mannheim, University of Heidelberg (2019-1085R). All patients signed informed consent and gave their approval for the use of clinical and radiographic data for scientific purposes. The conducted experiments respect the ethical standards in the Helsinki Declaration of 1975, as revised in 2000, as well as the national law.

Preoperative X-rays in 2 planes (anterior-posterior (AP) and Y-view) and a computed tomography (CT) scan of the affected side were obtained. All patients underwent surgery in beach chair position under general or regional anesthesia. Surgery was performed by one single surgeon (LL). For the implantation of the prostheses a deltopectoral approach was utilized in all cases.

### Subclassification

**Fig. 1:**
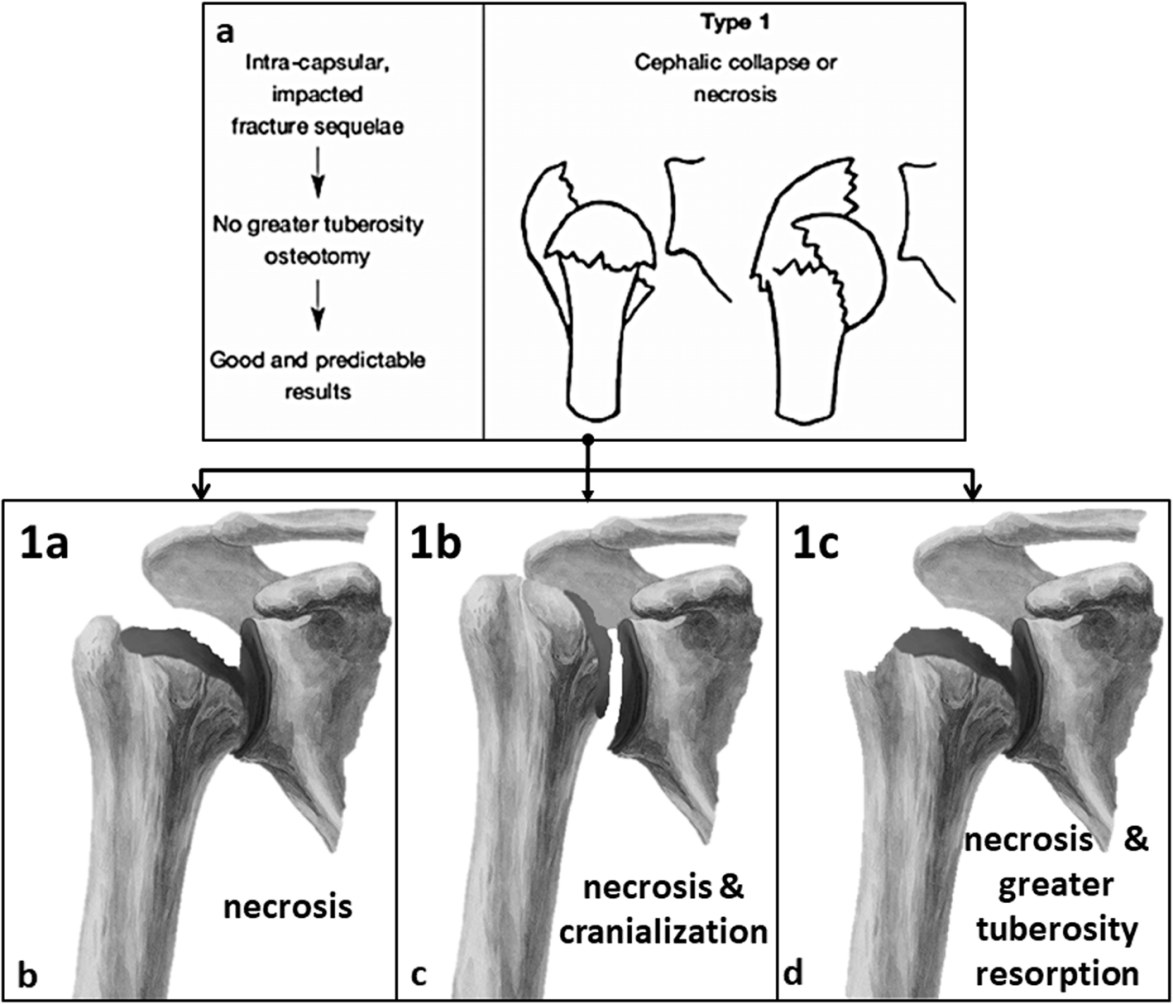
Subclassification of proximal humeral fracture sequelae type 1 according to Boileau [1] (**a**). Type 1 a lesions (**b**) present a humeral head necrosis without cranialization of the humeral head and can be treated with anatomic hemi or total shoulder arthroplasty. Type 1 b lesions (**c**) represent humeral head necrosis with cranialization of the humeral head (i.e. a reduced acromio-humeral interval < 7 mm) and should be treated with reverse shoulder arthroplasty. Type 1 c lesions (**d**) show humeral head necrosis with resorption of the greater tuberosity and should also be treated with reverse shoulder arthroplasty

All cases were classified according to the 3 suggested subtypes (Fig. 1). Type 1a lesions with posttraumatric osteonecrosis of the humeral head including varus and valgus deformities, type 1b lesions with posttraumatric osteonecrosis of the humeral head and a reduced AHI (< 7 mm) and type 1c lesions with posttraumatric osteonecrosis of the humeral head and additional resorption of the greater tuberosity.

### Surgical technique for subtype 1a

For type 1a lesions either a stemless hemiprosthesis or a stemless total shoulder prosthesis with a cemented keeled polyethylene glenoid component (dependent on the glenoid condition) was implanted (Eclipse; Arthrex, Naples, USA) according to the manufacturer’s instruction. Glenoid replacement was only performed in cases of osteoarthritis or large osteochondral defects of the glenoid fossa.

### Surgical technique for subtype 1b and 1c

For type 1b and 1c lesions a cementless reverse total shoulder prosthesis with 135° humeral inclination and with +4 mm glenosphere lateralization was implanted (Univers Revers; Arthrex, Naples, USA) according to the manufacturer’s instruction. If possible the subscapularis tendon was reattached after implantation of the prosthesis.

### Aftercare

Postoperatively the shoulder was usually immobilized in internal rotation for 6 weeks. Passive range of motion (ROM) was initiated at 3 weeks postoperative. The sling was removed at 6 weeks and active range of motion was allowed. Strengthening was allowed at 12 weeks postoperative.

### Postoperative evaluation

Data concerning characteristics of the patient at the moment of surgery, surgical technique, and complications were retrospectively retrieved from our institution’s electronic medical record system.

An independent observer examined all included patients and assessed the outcome of the procedure. For follow-up examination, the patients were asked to grade pain on a visual analogue scale (VAS). Active range of motion (ROM) was measured with a goniometer for elevation, abduction, and external rotation of the elbow at the side. Internal rotation was judged by the level of vertebra reached by the thumb. Functional outcome was assessed using the Constant-Murley score (CS). In addition, the Quick Disabilities of Shoulder and Hand (DASH) Score and the Subjective Shoulder Value (SSV) were used as a patient-focused outcome tools. In order to evaluate the patients’ general health condition the EQ. 5d score was used.

Classification according to Boileau was confirmed by consensus between three shoulder surgeons.

Radiographic assessment at follow-up was based on an AP view in neutral rotation and an axial view and was performed by one examiner (JS).

### Statistical analysis

Statistical analysis was performed with SPSS version 22 (IBM, Armonk, USA) using the independent samples Mann-Whitney U-test and the Kruskal-Wallis test. Quantitative variables were described by means, standard deviations, minimums and maximums. Normal distributions were tested by the Shapiro-Wilk test and confirmed graphically by histogram.

## Results

Of 69 patients with a FS of the proximal humerus 27 presented a FS type 1. 17 patients (63%) with a mean age of 64 ± 11 years were available for follow-up at 24 ± 10 months. 3 patients were deceased, 3 did not meet the inclusion criteria and 4 were unable to participate in the study. 3 of the cases occurred after conservative treatment and 14 cases after open reduction and internal fixation. 10 (59%) of the 17 patients were smokers or suffered from diabetes mellitus. Baseline characteristics are summarized in Table 1.

**Table 1:** Baseline characteristics

***Variable***	***Number***
Follow-up rate [percent]	17/27 [63%]
Mean patient age in years [SD]	64 [±11]
Mean follow-up in months [SD]	24 [±10]
Gender	
Male [percent]	3 [18%]
Female [percent]	14 [82%]
Injured side	
Right [percent]	10 [59%]
Left [percent]	7 [41%]
Fracture sequelae type 1 after	
Conservative treatment	3 [18%]
ORIF	14 [82%]
Radiographic parameters	
Preoperative acromio-humeral interval [mm]	9 [±3]
Greater tubersosity resorption [percent]	4 [24%]

*mm* Millimeter, *ORIF* Open reduction internal fixation, *SD* Standard deviation

One patient was treated with stemless total shoulder arthroplasty (Eclipse, Arthrex Inc., Naples, FL USA), 6 with stemless hemiarthroplasty (Eclipse, Arthrex Inc., Naples, FL USA) and 10 were treated with reverse shoulder arthroplasty (Univers Revers, Arthrex Inc., Naples, FL USA).

Mean postoperative active forward flexion was 110° ± 28°, mean abduction 98° ± 26° and mean external rotation at the side was 35° ± 10°. Mean internal rotation was at vertebra L3. CS improved significantly from 16 ± 7 points to 61 ± 19 points (*p* < 0.0001). At final follow-up the mean Quick DASH Score was 21 ± 21 and the mean SSV was 73 ± 21 points. Average pain level on the VAS was 1.9 ± 2.2 out of 10 points. The mean Eq. 5d general health score was 73 ± 17%.

The mean preoperative AHI was 9 ± 3 mm, however, 8 cases presented an AHI < 7 mm. In 4 cases the preoperative imaging showed complete greater tuberosity resorption. Retrospectively, those cases with a preoperative AHI < 7 mm or resorption of the greater tuberosity were analyzed regarding the chosen treatment method, i.e. reverse vs. anatomic arthroplasty. A subgroup analysis was performed and the postoperative results comparing these two treatment options are outlined in Table 2. In addition, the subgroup with humeral head necrosis including varus and valgus deformities is illustrated.

**Table 2:** Patient outcomes comparing those cases with an acromio-humeral interval ≤ 6 mm or greater tuberosity resorption treated with anatomic shoulder arthroplasty vs. reverse shoulder arthroplasty

	n	Constant [points]	Quick DASH [points]	SSV [%]	VAS Pain [points]	Forward flexion [°]	Abduction [°]	External rotation [°]	Internal rotation [°]
**AHI ≤ 6 mm**									
**- Anatomic**	**1**	**36**	**30**	**60**	**5.0**	**80**	**80**	**40**	**S1**
**- Reverse**	**7**	**60**	**25**	**75**	**2.0**	**106**	**94**	**36**	**L4**
**Greater tuberosity resorption**									
**- Anatomic**	**2**	**38**	**27**	**50**	**3.0**	**80**	**70**	**20**	**L5**
**- Reverse**	**3**	**56**	**23**	**77**	**2.7**	**97**	**87**	**27**	**L2**
**Head necrosis (including varus/valgus deformity)**									
**- Anatomic**	**4**	**77**	**11**	**74**	**0.5**	**143**	**125**	**43**	**L1**
**AHI ≤ 6 mm or greater tuberosity resorption**									
**- Reverse**	**10**	**59**	**23**	**75**	**2.2**	**103**	**92**	**33**	**L3**

In addition, results of patients with “normal” humeral head necrosis including varus and valgus deformity are illustrated. *AHI* acromio-humeral interval; *DASH* disabilities of shoulder and hand; *L* lumbar vertebra; S sacral vertebra; *SSV* subjective shoulder value; *VAS* visual analogue scale;

Those patients who presented either an AHI < 7 mm or resorption of the greater tuberosity and were nevertheless treated with ASA showed inferior postoperative results compared to those threated with RSA (Table 2). Patients with humeral head necrosis without reduced AHI or greater tuberosity resorption were treated with ASA and showed better clinical with a mean CS of 77 points compared to those treated with RSA in case of reduced AHI or greater tuberosity resorption (mean CS 59 points).

The overall complication rate was 19% and the revision rate was 19%; implant survival was 88%.

Two cases (1 ASA and 1 RSA case) presented a low-grade infection after 8 and 17 months. In both cases a 1-stage revision surgery with change of the prosthesis and 12 weeks of antibiotic treatment was performed. In another case a traumatic periprosthetic fracture type Worland C occurred in a patient with a reverse prosthesis and was treated with open reduction and internal fixation [9].

The 3 patients who suffered a complication showed significantly inferior functional results compared to the rest of the cohort (mean CS 34 vs. 64 points; *p* = 0.015).

## Discussion

Fracture sequelae of the proximal humerus are rare pathologies and were first classified by Boileau et al. [1, 2] This classification devides FS of the proximal humerus in four types divided into two categories. FS of category I represent intracapsular pathologies and are divided into type 1 lesions with humeral head necrosis, and type 2 sequelae including locked dislocations or fractured dislocations. However, in 2012, Moineau et al. [5] realized that FS type 1 should not always be treated with ASA and therefore divided type 1 sequelae in four groups: 1A – isolated posttraumatic osteonecrosis of the humeral head without tuberosity malunion; 1B – isolated posttraumatic osteoarthritis without osteonecrosis or tuberosity malunion; 1C – proximal humeral deformity with valgus malunion secondary to valgus impacted fracture; 1D – varus malunion secondary to varus impacted fracture. In 55 patients after a mean follow-up of 52 months significantly poorer results were associated with varus deformity and with fatty infiltration of the rotator cuff. In this cohort, proximal humeral deformity, i. e. varus or valgus malunion of the greater tuberosity, was related to inferior CS values of 10 points and decreased active elevation of almost 20° compared to patients with no such deformity. The poorest results were observed in cases of varus malunion. They also found that patients with a postoperative acromio-humeral distance of <7 mm had significantly poorer results than patients with a distance of >7 mm. Therefore, they recommended using a RSA in patients with these factors but had no data to support this suggestion.

Tauber et al. [10] published the results of a case series of 38 patients with traumatic humeral head necrosis and focused on the influence of preoperative greater tuberosity position and tuberosity resorption. In patients with resorption of either the lesser or greater tuberosity, the outcome after anatomic humeral head replacement was unsatisfying. The most relevant factor was restricted range of motion. None of these patients achieved a CS superior to 51%.

Several studies have outlined the importance of the greater tuberosity in the treatment of FS type 1. In patients with malunion with the need for greater tuberosity osteotomy, the final outcome after humeral head replacement was impaired and linked to unpredictable results [11].

Similarly, a significant difference was reported in a cohort treated with shoulder ASA for posttraumatic changes after proximal humeral fracture between those who required greater tuberosity osteotomy and those who did not [1].

In the subgroup of patients who suffered intracapsular lesions and did not require greater tuberosity osteotomy, the postoperative results were good to excellent. In contrast, in cases requiring osteotomy and repositioning of the greater tuberosity for implantation of the prosthesis, outcome was poor and none of those patients achieved an active flexion beyond 90°.

As RSA became more popular in the last decade, several studies reported the use of RSA for the treatment of FS type 1.

Willis et al. described 16 patients with proximal humeral FS, whereas six were type 1 with preoperative fatty degeneration of the rotator cuff [12]. Gwinner et al. found a significant increase of the postoperative CS (15 points preoperatively vs. 54 points) in ten patients with type 1 FS and bone deficiencies or fatty infiltration of the rotator cuff [13]. Martinez et al. [14] examined a cohort of 44 patients with different types of FS of the proximal humerus, 16 were type 1 FS. Subgroups were not analyzed; however, a high complication rate of 27% was reported. Raiss et al. [4] reported a multicenter study with 38 cases of type 1 FS of the proximal humerus in association with rotator cuff deficiency or severe stiffness of the shoulder. The mean CS improved from 25 points preoperatively to 57 points postoperatively. In contrast to varus and valgus deformities, rotator cuff tears and stiffness of the shoulder had an adverse effect on the clinical outcome. Further studies examined RSA as a salvage procedure after previous internal fixation of proximal humeral fractures and showed satisfying clinical results with a relatively low complication rate [15–17].

More recently, two comparative studies for the treatment of FS with ASA and RSA have been published.

Kilic et al. [18] reviewed the results of 55 patients with FS of the humeral head, 36 cases with ASA and 19 with RSA. In the ASA group, 32 had a type 1 or 2 sequelae and 4 a type 3 or 4. In the RSA group, 2 patients had a type 1 or 2 sequelae, and 17 a type 3 or 4. After ASA the mean Constant scores improved from 19 to 68 points for FS type 1 and 2, and from 9 to 47.5 points after RSA for FS type 3 and 4. The authors confirmed the results and indications proposed by Boileau et al. [1] and concluded that for FS type 1 ASA is the better choice. However, they did not further analyze and compare those patients with FS type 1 treated with ASA and RSA.

Alentorn-Geli et al. [19] realized a comparative study analyzing the results of 12 hemiarthroplasties and 20 RSA for the treatment of FS. In the hemiarthroplasty group there were six type 1 sequelae, two type 2, and four type 4. In the RSA group there were four type 1, three type 2, three type 3, and ten type 4. At follow-up there were no significant differences between both groups. However, there were more complications in the hemiarthroplasty group compared to RSA. One case required conversion to an anatomic total shoulder prosthesis because of glenoid erosion, one case conversion to a reverse prosthesis because of pain and functional limitations, and one case conversion to RSA after infection.

The results of our study regarding functional outcome and patient satisfaction are comparable to the above mentioned studies.

Nevertheless, none of the above studies compared the results of ASA and RSA only for FS type 1. In our cohort we could show that good and reproducible clinical results can be achieved with ASA if there is neither greater tuberosity resorption nor an AHI smaller than 7 mm. In 3 cases (1 with AHI < 7 mm and 2 with greater tuberosity resorption) we performed ASA and functional outcome was poor. Unfortunately, due to the small case number it is not possible to make a statistically meaningful statement. At the 2 year follow-up all 3 patients stated that they were prone to a revision surgery with conversion to RSA.

**Fig. 2:**
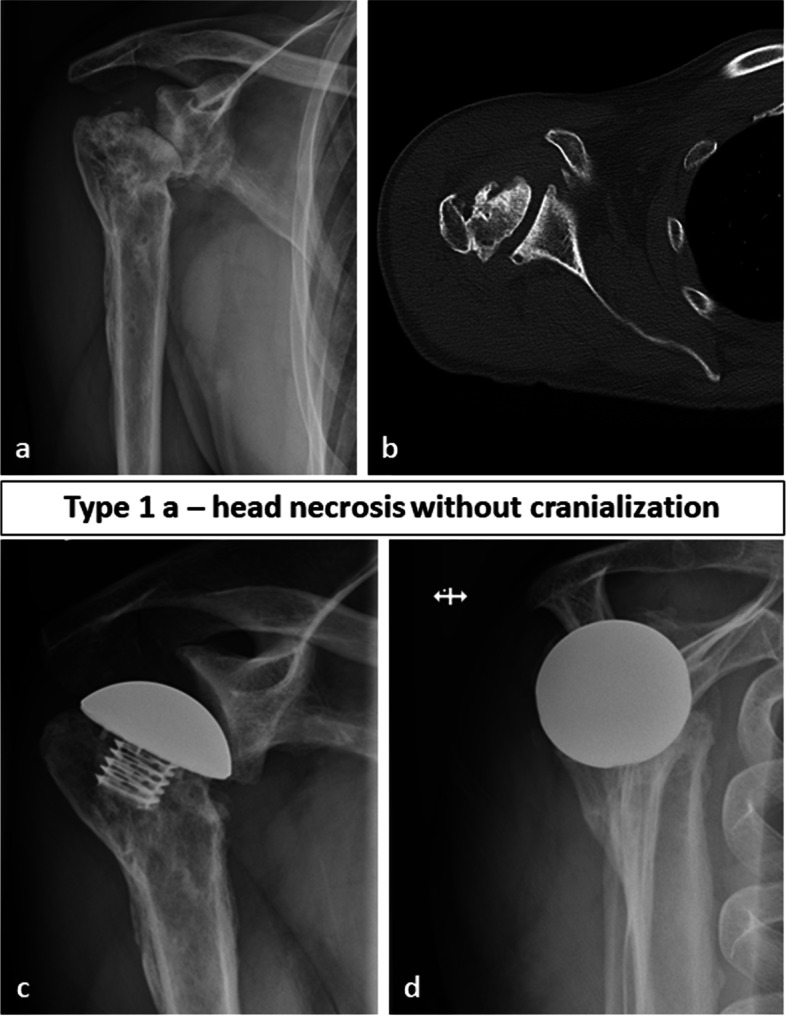
Preoperative (**a**, **b**) and follow-up images (**c**, **d**) of a 49-year-old male patient with a fracture sequelae of the proximal humerus type 1 a i.e. a humeral head necrosis without cranialization of the humeral head treated with anatomic stemless hemiarthroplasty. At final follow-up after 24 months the Constant Score was 77 points

**Fig. 3:**
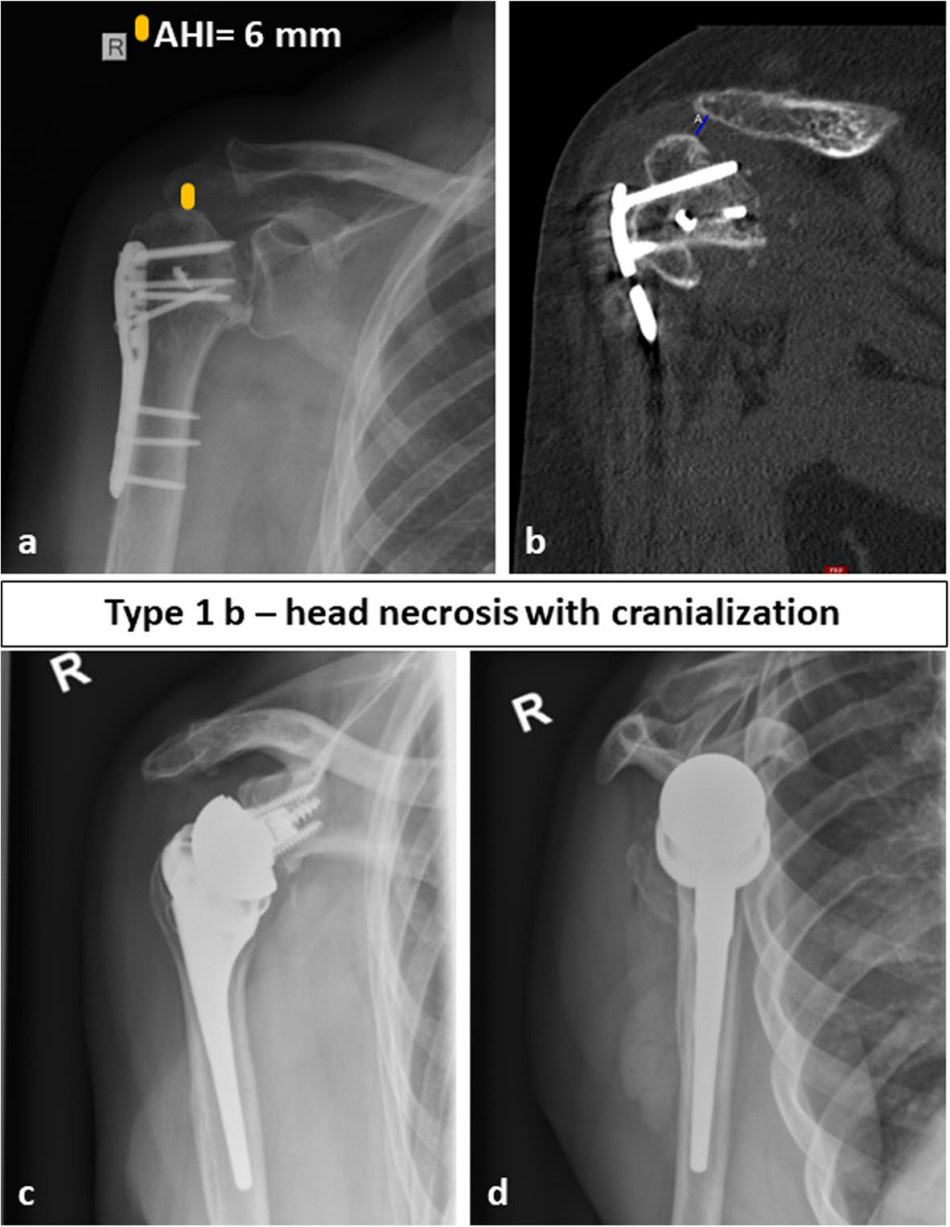
Preoperative (**a**, **b**) and follow-up images (**c**, **d**) of a 70-year-old female patient with a fracture sequelae of the proximal humerus type 1 b i.e. a humeral head necrosis with cranialization of the humeral head treated with reverse shoulder arthroplasty. At final follow-up after 28 months the Constant Score was 56 points

**Fig. 4:**
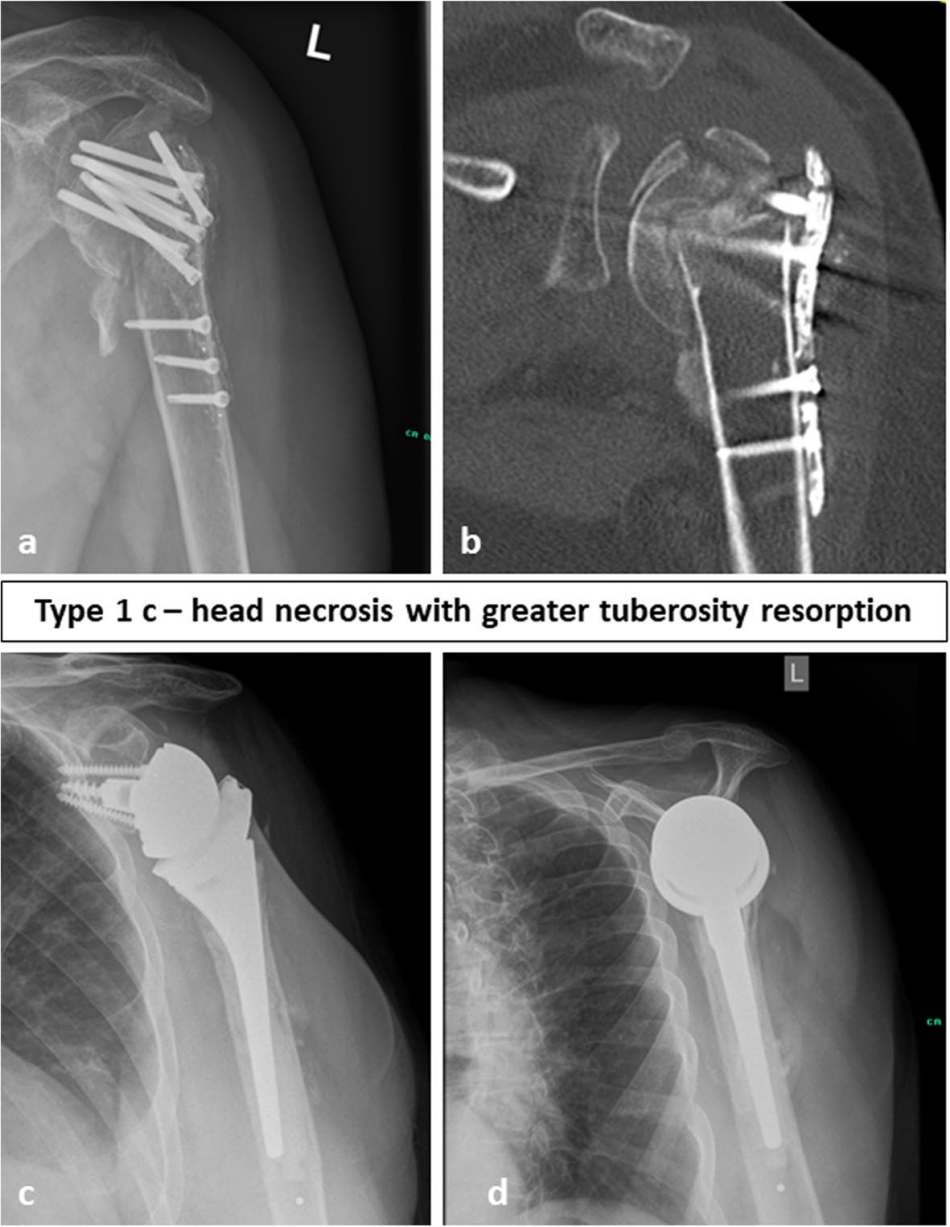
Preoperative (**a**, **b**) and follow-up images (**c**, **d**) of a 75-year-old female patient with a fracture sequelae of the proximal humerus type 1 c i.e. a humeral head necrosis with resorption of the greater tuberosity treated with reverse shoulder arthroplasty. At final follow-up after 20 months the Constant Score was 72 points

Thus, in our eyes, the classification of fracture sequelae according to Boileau is insufficient for type 1 lesions and the general treatment recommendation with ASA has to be reconsidered. Moineau et al. [5] already realized this and divided FS type 1 in 4 different subgroups, however, these subgroups only have limited influence on the decision-making for the right treatment option. With his classification for FS Boileau tried to guide treatment, therefore, we suggest a new subclassification system for FS type 1 with 3 subtypes as shown in Fig. 1 (Fig. 1): type 1a lesions with posttraumatric osteonecrosis of the humeral head including varus and valgus deformities, type 1b lesions with posttraumatric osteonecrosis of the humeral head and a reduced AHI (< 7 mm) and type 1c lesions with posttraumatric osteonecrosis of the humeral head and additional resorption of the greater tuberosity. Exemplary cases are shown in Figs. 2, 3 and 4. As we could show type 1a lesions can be treated with ASA with good clinical results, however, type 1b and 1c lesions, i.e. those with dysfunction of the rotator cuff, should be treated with RSA in order to achieve consistent postoperative results.

Our study cohort was consistent with the literature regarding the complication rate: all reported fracture sequelae complication rates ranged from 16 to 41%, and revision rates ranged from 11 to 41% [2, 5, 15, 20, 21]. It is obvious that infection is one of the main concerns as many patients underwent previous shoulder surgery for treatment in the acute fracture situation.

### Limitations and strengths

There are several limitations to our study. It is a retrospective study without a control group. These clinical outcomes correspond to our initial experience, sample size is therefore small, and we report our short-term results. No complications or revisions of the patients who were lost to follow-up or deceased were documented. Further evaluation should be considered to confirm our recommendations.

A major strength of the study is that only type I FS were included. Most other studies the treatment of FS report different types of sequelae assembled in a single cohort of patients. In addition, a single experienced shoulder surgeon performed all operations. Therefore, no bias caused by different surgeons with varying experience has to be taken into account when the results.

## Conclusion

With good preoperative planning and by using the adequate surgical technique good clinical short-term results with a relatively low complication rate can be achieved in cases with FS type 1. The Boileau classification should be extended for fracture sequelae type 1 and the general recommendation for treatment with hemiarthroplasty or total shoulder arthroplasty has to be relativized. Special attention should be paid to a decreased AHI and/or resoprtion of the greater tuberosity as indirect signs for dysfunction of the rotator cuff. To facilitate the choice of the adequate prosthetic treatment method the suggested subclassification system, which facilitates the indication for anatomic and reverse shoulder arthroplasty, should be applied.

## Data Availability

The datasets used and/or analysed during the current study are available from the corresponding author on reasonable request as participants of this study did not agree for their data to be shared publicly.

## Abbreviations

AHI: Acromio-humeral interval
AP: Anterior-posterior
ASA: Anatomic shoulder arthroplasty
CS: Constant score
CT: Computed tomography
DASH: Disabilities of shoulder and hand
FS: Fracture sequelae
ROM: Range of motion
RSA: Reverse shoulder arthroplasty
SSV: Subjective shoulder value
VAS: Visual analogue scale
